# Errata

**Published:** 2005-05

In Table 1 of “Estimating the Exposure–Response Relationships between Particulate Matter and Mortality within the APHEA Multicity Project” by Samoli et al. [ Environ Health Perspect 113:88–95 (2005)], the values for CVD deaths are incorrect. The corrected table is shown below. The authors apologize for the errors.

Bonner et al. would like to correct a factual error in “Occupational Exposure to Carbofuran and the Incidence of Cancer in the Agricultural Health Study” [ Environ Health Perspect 113:285–289 (2005)]. In the second paragraph of the introduction, the fourth sentence was incorrect. The two studies cited demonstrated that another carbamate pesticide, carbendazim, and not carbofuran, induced lymphoma. The sentence should read: “While two studies demonstrated that the carbamate pesticide carbendazim was able to induce lymphoma in Swiss mice (Borzsonyi and Pinter 1977; Borzsonyi et al. 1976), carcinogenicity of carbofuran was not evident in several 2-year dietary studies conducted on rats (Gupta 1994).”

Despite the error, the authors stand by the validity of the analysis and the interpretation of the results. The authors apologize for the error.

The March Focus article [“Great Lakes: Resource at Risk,” Environ Health Perspect 113:A164–A173 (2005)] stated that Dow Chemical released about 400 tons of mercury into Lake Superior from two chloralkali plants. In fact, these two plants were located in Sarnia, Ontario. Thus, the discharges were made into Lake Huron. *EHP* regrets the error.

## Figures and Tables

**Table 1:** City descriptive data on the study period, population, exposure (PM_10_ and BS), outcome (daily number of deaths), and selected effect modifiers (region, mean temperature, mean NO_2_ over 24 hr, and directly standardized mortality rate).

			No. of deaths per day			PM_10_ (μg/m^3^) percentile		BS (μg/m^3^) percentile					
City	Study period (month/year)	Population (×1,000)	Total	CVD	Respiratory	50th	90th	50th	90th	Geographic region	Mean temperature	NO_2_ (24-hr)	SDR
Athens	1/92–12/96	3,073	73	36	5	40*^a^*	59	64	122	South	18	74	784
Barcelona	1/91–12/96	1,644	40	16	4	60	95	39	64	South	16	69	740
Basel	1/90–12/95	360	9	4	1	28*^a^*	55			West	11	38	678
Bilbao	4/92–3/96	667	15	5	1			23	39	South	15	49	711
Birmingham	1/92–12/96	2,300	61	28	9	21	40	11	22	West	10	46	895
Budapest	1/92–12/95	1,931	80	40	3	40*^a^*	52			East	11	76	1,136
Cracow	1/90–12/96	746	18	10	0	54*^a^*	86	36	101	East	8	44	1,009
Dublin	1/90–12/96	482	13	6	2			10	26	West	10	—	940
Erfurt	1/91–12/95	216	6	—	—	48	98			West	9	40	972
Geneva	1/90–12/95	317	6	2	0	33*^a^*	71			West	10	45	608
Helsinki	1/93–12/96	828	18	9	2	23*^a^*	49			West	6	33	915
Ljubljana	1/92–12/96	322	7	3	0			13	42	East	11	46	823
Lodz	1/90–12/96	828	30	17	1			30	77	East	8	39	1,231
London	1/92–12/96	6,905	169	71	29	25	46	11	22	West	12	61	851
Lyon	1/93–12/97	416	9	3	1	39	63			West	12	63	579
Madrid	1/92–12/95	3,012	61	22	6	33	59			South	15	70	636
Marseille	1/90–12/95	855	22	8	2			34	56	West	16	71	666
Milan	1/90–12/96	1,343	29	11	2	47*^a^*	88			West	14	94	632
Netherlands	1/90–9/95	15,400	342	140	29	34	67	63	122	West	10	43	757
Paris	1/92–12/96	6,700	124	38	9	22	46	21	45	West	12	53	644
Poznan	1/90–12/96	582	17	9	1			23	76	East	9	47	1,106
Prague	2/92–12/95	1,213	38	22	1	66	124			East	10	58	984
Rome	1/92–12/96	2,775	56	23	3	57*^a^*	81			South	17	88	585
Stockholm	1/94–12/96	1,126	30	15	3	14	27			West	8	26	666
Tel Aviv	1/93–12/96	1,141	27	12	2	43	75			South	20	70	430
Teplice	1/90–12/97	625	18	10	1	42	83			East	9	32	1,173
Torino	1/90–12/96	926	21	9	1	65*^a^*	129			West	14	76	724
Valencia	1/94–12/96	753	16	6	2			40	70	South	19	66	820
Wroclaw	1/90–12/96	643	15	9	1			33	97	East	9	27	970
Zurich	1/90–12/95	540	13	6	1	28*^a^*	54			West	11	40	666

Abbreviations: —, no data; CVD, cardiovascular deaths; SDR, directly standardized mortality rate. Mean temperature in degrees centigrade.

*^a^*PM_10_ were estimated using a regression model relating collocated PM_10_ measurements to the BS or total suspended particles.

